# Min pig skeletal muscle response to cold stress

**DOI:** 10.1371/journal.pone.0274184

**Published:** 2022-09-26

**Authors:** Dongjie Zhang, Shouzheng Ma, Liang Wang, Hong Ma, Wentao Wang, Jiqao Xia, Di Liu

**Affiliations:** 1 Institute of Animal Husbandry, Heilongjiang Academy of Agricultural Sciences, Harbin, Heilongjiang, People’s Republic of China; 2 Department of Animal Science, Northeast Agricultural University, Harbin, Heilongjiang, People’s Republic of China; Central University of Punjab, INDIA

## Abstract

The increased sensitivity of pigs to ambient temperature is due to today’s intensive farming. Frequent climate disasters increase the pressure on healthy pig farming. Min pigs are an indigenous pig breed in China with desirable cold resistance characteristics, and hence are ideal for obtaining cold-resistant pig breeds. Therefore, it is important to discover the molecular mechanisms that are activated in response to cold stress in the Min pig. Here, we conducted a transcriptomic analysis of the skeletal muscle of Min pigs under chronic low-temperature acclimation (group A) and acute short cold stress (group B). Cold exposure caused more genes to be upregulated. Totals of 125 and 96 differentially expressed genes (DEGs) were generated from groups A and B. Sixteen common upregulated DEGs were screened; these were concentrated in oxidative stress (*SRXN1*, *MAFF*), immune and inflammatory responses (*ITPKC*, *AREG*, *MMP25*, *FOSL1*), the nervous system (*RETREG1*, *GADD45A*, *RCAN1*), lipid metabolism (*LRP11*, *LIPG*, *ITGA5*, *AMPD2*), solute transport (*SLC19A2*, *SLC28A1*, *SLCO4A1*), and fertility (*HBEGF*). There were 102 and 73 genes that were specifically differentially expressed in groups A and B, respectively. The altered mRNAs were enriched in immune, endocrine, and cancer pathways. There were 186 and 91 differentially expressed lncRNAs generated from groups A and B. Analysis of the target genes suggested that they may be involved in regulating the MAPK signaling pathway for resistance to cold. The results of this study provide a comprehensive overview of cold exposure–induced transcriptional patterns in skeletal muscle of the Min pig. These results can guide future molecular studies of cold stress response in pigs for improving cold tolerance as a goal in breeding programs.

## Introduction

Low-temperature cold waves are one of the most common natural disasters that can have destructive effects on animals, plants, and microorganisms. Although domestic livestock and poultry are artificially protected, meaning that the degree of damage can be reduced, the current large-scale breeding model makes livestock and poultry more sensitive to ambient temperature. Pigs are one of the most important livestock animals in the world. Compared with other large livestock species such as cattle and sheep, they are more sensitive to changes in ambient temperature [1]. The critical temperatures of pigs at various growth stages are significantly different, being 26–28°C for weaning piglets and 18–20°C for fattening pigs weighing 60 kg [2]. Below these temperature ranges, pigs are regarded as being in a low-temperature environment. A low-temperature environment is the main cause of death and morbidity of newborn piglets [3], a phenomenon that increases the mortality rate of sows in the perinatal period [4] and decreases meat quality [5]. Therefore, the relationship between pigs and ambient temperature is one of the issues that cannot be ignored in the pig breeding industry.

Over the past 20 years, rapid advances in sequencing technology have enabled the study of genome-wide patterns of gene expression changes in response to various internal and external stimuli, and low-temperature stress has been found to have broad effects on an organism’s transcriptome. In deep sequencing of an Antarctic bacterium, it was found that genes related to primary metabolism were inhibited at low temperature, while genes related to transcriptional regulatory proteins and signal transduction proteins were promoted, and the ethanol oxidation pathway was effective for bacterial growth at low temperatures [6]. Genome-wide analysis of low temperature tolerance in grapefruit revealed downregulation of photosynthesis, cell wall synthesis, and secondary metabolism-related transcripts, while there was upregulation of membrane proteins, lipid metabolism, plant hormones, and cold-responsive transcription factors [7]. Short-term cold exposure (3d, 4°C) altered gene expression and biological pathways involved in fatty acid elongation and triacylglycerol, sphingolipid, and triglyceride synthesis in inguinal white fat (iWAT) of mice; in addition, along with metabolic syndrome, neurodegenerative diseases and aging-related genes and expression pathways will also undergo changes [8]. The expression levels of 91 mRNAs in the iliopsoas muscle of rats subjected to severe hypothermia were more than twice those of the control group, and these mRNAs were involved in a series of biological processes, including responses to stress and lipids and cellular responses to hypoxia. *CTGF*, *JUNB*, *NR4A1*, and *SDC4* were genes specifically expressed under severe hypothermia [9]. Transcriptome sequencing of human vastus lateralis muscle found that cold acclimation changed the expression levels of 756 genes, and the genes with the highest expression level changes were involved in the contraction and signal transduction between nerve cells and muscle cells. There is significant overlap between genes and the upregulation of genes caused by exercise training, especially genes and pathways related to extracellular matrix remodeling [10].

When the body is stimulated by low temperature, it first maintains a constant body temperature through vasoconstriction and shivering heat production. At this time, skeletal muscle contraction and heat production play an important role. However, with the prolongation of low temperature time, the body gradually changed from shivering thermogenesis to non-shivering thermogenesis. At this time, the metabolic thermogenesis of fat was the main one, especially the metabolic thermogenesis of brown fat. Bal et al. found that during mild and severe cold stimulation, non-shivering thermogenesis of muscle was initiated simultaneously with metabolic thermogenesis of brown fat, and if one pathway was blocked, the other pathway increased thermogenesis to maintain core temperature [11]. In addition, mitochondrial crosstalk in the sarcoplasmic reticulum, increased mitochondrial biosynthesis, uncoupling protein 3 (UCP3)-induced thermogenesis, and a key enzyme in gluconeogenesis, fructose-1,6-bisphosphatase (Fructose 1), 6-bisphosphatase 2, FBP2), will affect the non-shivering thermogenesis of skeletal muscle [12,13]. Skeletal muscle plays an important role in maintaining a constant body temperature in homeothermic animals. However, due to limitations of the experimental conditions, there are few research reports concerning large domestic animals.

Min pigs are an indigenous breed distributed in northeastern China. The breed has remarkable cold-resistant characteristics [14], and can maintain normal physiological responses in a wide temperature range, from −30°C to over 30°C. The aims of this study were to screen and identify the genes and regulatory factors involved in low-temperature stress response in Min pig skeletal muscle and to reveal the molecular mechanisms of cold tolerance. We performed RNA-Seq analysis on Min pig skeletal muscle tissue from Min pigs subjected to hypothermia for three days and 58 days.

## Materials and methods

### Ethics statement

We have followed EU standards for the protection of animals used for scientific purposes. The experimental protocols used in this study were reviewed and approved by the Animal Care and Use Committee of Heilongjiang Academy of Agricultural Sciences, People’s Republic of China.

### Animal experiment design

The experiment was carried out at the Min Pig Conservation Farm of Institute of Animal Husbandry, Heilongjiang Academy of Agricultural Sciences, China. The experiment lasted 58 days, from November to December 2020.

Nine six-month-old female Min pigs with similar body weights were randomly divided into three groups, with three pigs in each group. Before the start of the experiment, all Min pigs were raised in a heated pig house, and the temperature was controlled at (18 ± 2)°C. When the experiment started, three Min pigs (group A: the chronic low-temperature acclimation group) were chased outside. At that time, the environmental temperature range for the full day was 5°C/−5°C (highest temperature/lowest temperature); after 55 days, the ambient temperature dropped to −15°C/−24°C (highest temperature/lowest temperature). The temperature transformation is shown in S1 Fig. On the 55^th^ day, another group (group B: the acute short cold stress group) were also chased outside. The remaining individuals remained in a heated pig house (group C: the negative control group). Three days later, all individuals were slaughtered by electric shock (110 volts, head electric shock), and 100 mg of the longissimus dorsi muscle of each pig was taken and stored in liquid nitrogen for future use. During the experiment, all individuals were given free access to food and water.

### RNA extraction and RNA-seq

Total RNA of skeletal muscle was extracted from each pig (n = 3 for groups A, B, and C) using the Trizol reagent. The purity of the extracted RNA was assayed by a Nanodrop 2000 spectrophotometer (Thermo Fisher, Waltham, MA, USA), and the concentration and integrity of total RNA were tested by using an Agilent 2100 RNA Nano 6000 Assay Kit (Agilent Technologies, Palo Alto, USA). The final required concentration of total RNA was not less than 100 ng/μL, and the ratio 28S/18S was ≥ 1.5. Before sequencing, rRNA was separated from total RNA. The cDNA library of each individual was constructed and sequenced on an Illumina NovaSeq 6000 platform (Illumina, Santiago, USA). Each read generated lengths of 150 bp by paired-end sequencing.

### Analysis of sequencing data

All profiling work for sequencing data was performed with the assistance of Annoroad Gene Technology Co., Ltd. (Beijing, China). Briefly, the raw sequencing data were evaluated by FAST-QC, including filtering of raw data, evaluation of the sequencing error rate distribution, and measurement of the GC content distribution. The clean reads were aligned to the *Sus scrofa* Ensembl 97 genome by the HISAT2 (http://ccb.jhu.edu/software/hisat2) program. Read counts for each gene in each sample were obtained by HTSeq, and FPKM (Fragments Per Kilobase per Million Mapped Reads) was then calculated to represent the expression level of each gene in the sample. DESeq2 was employed for differential gene expression analysis between groups. DESeq2 estimates the expression level of each gene in a sample by linear regression, then calculates the p-value via Wald’s test. Finally, the p-value was corrected by the BH method. Genes with q ≤ 0.05 and |log_2_ FC| ≥ 1 were identified as differentially expressed genes (DEGs). Volcano plots based on the DEGs analyses were drawn using R.

### Identification of lncRNA

The screening conditions for lncRNAs were as follows. The length of the transcript should be greater than or equal to 200 bp, and the number of exons should be greater than or equal to two. The reads coverage of each transcript was calculated, and the transcripts with fewer than five reads in all samples were screened out. The GffCompare (http://ccb.jhu.edu/software/stringtie/gff.shtml) program was used to make comparisons with pig annotation files to screen out known mRNAs and other non-coding RNAs (rRNA, tRNA, snoRNA, and snRNA) in pigs. The potential lincRNAs, intronic lncRNAs, and anti-sense lncRNAs were screened according to the class_code information (“u,” “i,” “x”). CNCI, CPC, CPAT, and PFAM protein domain analysis software programs were integrated to screen for coding potential. Only transcripts that were simultaneously judged as non-coding sequences by the four software programs were identified as lncRNAs.

### GO and KEGG pathway enrichment analysis

To investigate whether genes from each GO (Gene Ontology, http://geneontology.org/) term were enriched, a hypergeometric p-value was calculated and adjusted as a q-value, where the background was set as the genes of the whole genome. GO terms with q < 0.05 were considered to be significantly enriched. GO enrichment analysis identifies the biological functions of DEGs. KEGG (Kyoto Encyclopedia of Genes and Genomes, http://www.kegg.jp/) is a database resource that contains a collection of manually drawn pathway maps representing our knowledge of molecular interaction and reaction networks. Significantly enriched KEGG pathways were identified using the same method as in the GO enrichment analysis.

### The co‐expression network of lncRNA and mRNA

The mRNAs with a high Spearman correlation coefficient (r ≥ 0.9) were regarded as the trans-targets of lncRNAs, and the mRNAs within a distance of less than 50 kb were selected as the Cis-targets of lncRNAs. To identify interactions among the differentially expressed lncRNAs and mRNAs, we constructed a co-expression network based on a correlation analysis of the differentially expressed lncRNAs and mRNAs. The co-expression network was constructed based on the coexisting differentially expressed mRNAs between groups A and C, and groups B and C, and the lncRNAs that may regulate those mRNAs. The lncRNA–mRNA co-expression network was constructed using Cytoscape software. The target genes of differentially expressed lncRNAs were analyzed by functional enrichment, similar to the differential mRNA enrichment analysis. Bioinformatic analysis was performed using the OmicStudio tools at https://www.omicstudio.cn/tool.

### qRT-PCR validation

Total RNA was extracted and isolated from the samples that were consistent with the sequenced samples. RNA was reverse transcribed to cDNA using a PrimeScript^TM^ RT reagent Kit with gDNA Eraser (Takara, Kyoto, Japan) and stored at −20°C. Primer sequences are listed in S1 Table. The qRT-PCR amplification was performed under the following conditions: denaturing at 95°C for 10 min, 40 amplification cycles of denaturation at 95°C for 15 s, annealing at 60°C for 30 s, and extension at 72°C for 30 s, followed by acquisition of a fluorescence signal. The qRT‐PCR was performed with an SYBR Premix Ex Taq II (Takara, Kyoto, Japan), and completed by using a LightCycler 480 II Real‐Time PCR Detection System (Roche, Basel, Switzerland). The 2^-ΔΔ*C*t^ method was used to calculate the relative fold change of RNA expression. The mean Ct values were calculated from triplicate technical replicates.

## Results

### Identification of differentially expressed mRNAs during cold environment

**Fig 1:**
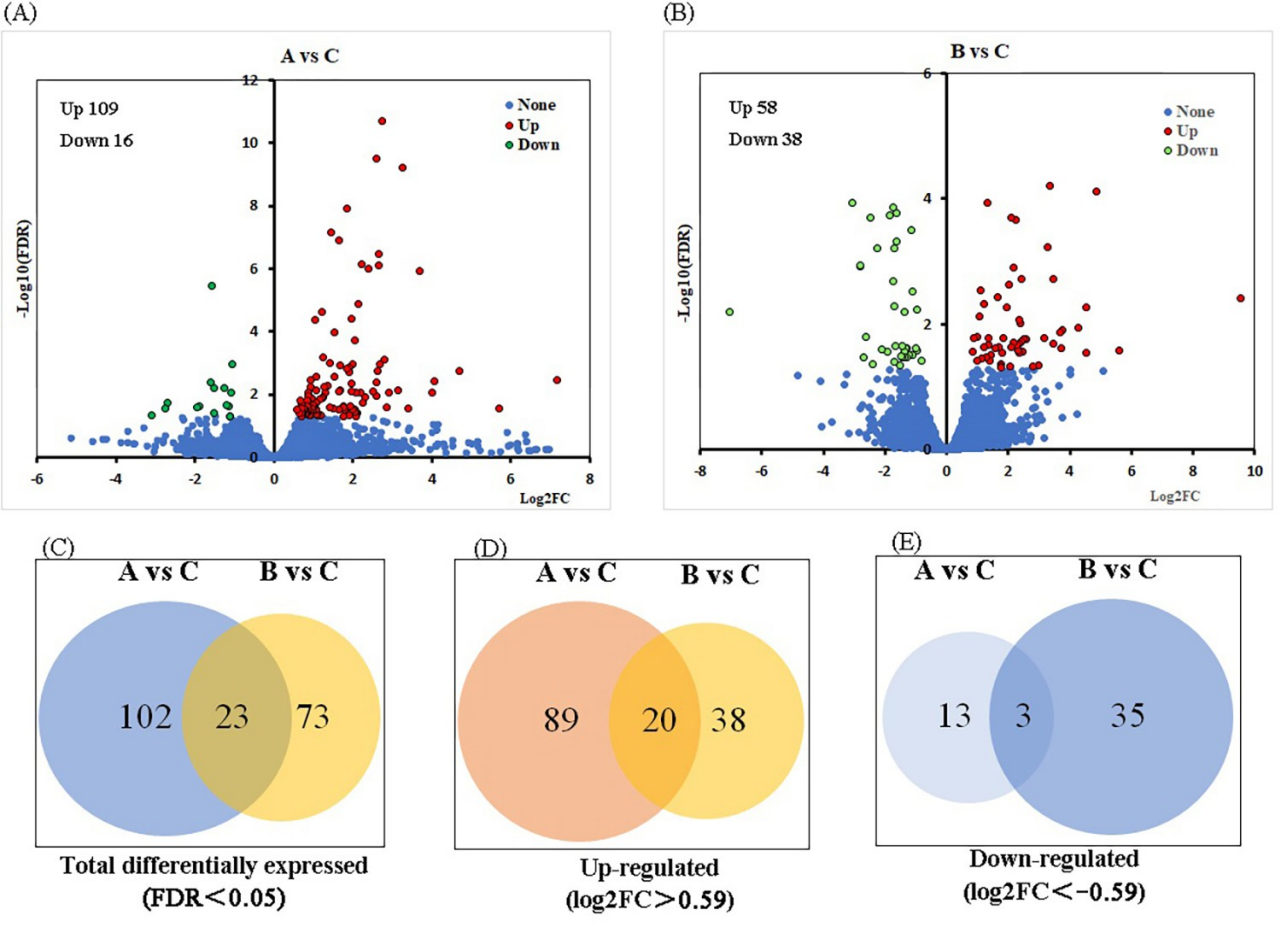
DEGs in the skeletal muscle of Min pig after cold stress. A. Volcano plot for chronic low-temperature acclimation group (A) after cold stress. Red points represent the upregulated genes; green points represent the downregulated genes (|log_2_FC|>0.59 and p< 0.05), and blue points represent the not significantly DEGs (p> 0.05). B. Volcano plot for acute short cold stress group (B) after cold stress. The means of color points are similar to A. C. Total DEGs in the groups A and B after cold stress. D. Upregulated DEGs in the groups A and B after cold stress. E. Downregulated DEGs in the groups A and B after cold stress.

To identify new molecular mechanisms of skeletal muscle when the body is placed in a cold environment, nine Min pigs were randomly selected from the six‐month-old pig group. Three pigs were assigned to chronic low-temperature acclimation (A); three pigs were defined as an acute short cold stress group (B), and three pigs were defined as a negative control group (C). The skeletal muscle of the above pigs was preserved in liquid nitrogen, and total RNA was used for whole‐transcriptome analysis via RNA‐seq. The clean reads were mapped to the reference genome and were spliced into putative transcripts (S2 Table). The bioinformatic analysis was divided into two independent comparisons: chronic low-temperature acclimation vs. the negative control group (A vs. C) and the acute short cold stress group vs. the negative control group (B vs. C). Volcano plots and heat map provided overview of the differential expression of mRNAs (Figs 1A, 1B and S2).

An absolute log_2_FC cutoff value of 0.59 and q < 0.05 were utilized as the criteria to confirm downregulated and upregulated mRNAs. Using these standards, there were 109 upregulated mRNAs and 16 downregulated mRNAs in A vs. C, and 58 upregulated mRNAs and 38 downregulated mRNAs were verified in B vs. C (S3 and S4 Tables). More mRNAs were altered in the gradual long cold stress period, while more mRNAs experienced downregulation under acute short cold stress. It has been speculated that there are differences in the gene expression profiles under different degrees of low-temperature stress. Venn diagrams revealed common and unique differentially expressed mRNAs in A vs. C and B vs. C. Compared to group C, 102 DEGs were expressed only in group A, and 73 DEGs were expressed only in group B. Twenty-three mRNAs had consistent expression changes between the two groups, including 20 upregulated mRNAs and three downregulated mRNAs. According to the number of downregulated mRNAs, we speculated that the acute short cold stress inhibited the transcription of many genes, but with time the transcription levels of some mRNAs returned to normal. Upregulated mRNAs of group A were more numerous than in group B, suggesting that low temperature activates the transcription of many genes. This demonstrated that the expression profiles of skeletal muscle under chronic low-temperature acclimation or acute short cold stress are different (Fig 1C–1E).

### The altered mRNAs were primarily enriched in immune, endocrine, and cancer pathways

After cold stress, the altered mRNAs with upregulated expression of chronic low-temperature acclimation group were enriched in biological processes, comprising 43 terms sorted by q values. The top five most significant terms were Cellular response to insulin stimulus (GO:0032869), Negative regulation of mitotic DNA damage checkpoint (GO: 1904290), CD4-positive, alpha-beta T cell lineage commitment (GO: 0043373), Enkephalin processing (GO: 0034230) and Negative regulation of follicle-stimulating hormone secretion (GO:0046882) (S5 Table). In the acute short cold stress group, the top five most significant terms were Negative regulation of activation of membrane attack complex (GO: 0001971), Cytidine transport (GO: 0015861), Positive regulation of protein targeting to mitochondrion (GO: 1903955), Nucleoside transmembrane transport (GO: 1901642) and Uridine transport (GO: 0015862) (S6 Table). The altered mRNAs with downregulated expression of chronic low-temperature acclimation group were enriched in biological process, including four terms sorted by q value: Maternal process involved in parturition (GO: 0060137), Regulation of systemic arterial blood pressure by vasopressin (GO: 0001992), Female pregnancy (GO: 0007565) and Positive regulation of vasoconstriction (GO: 0045907) (S7 Table). In the acute short cold stress group, 23 terms were enriched. The top five terms were Cellular response to thyrotropin-releasing hormone (GO: 1905229), Cellular response to glycoprotein (GO: 1904588), Thyroid-stimulating hormone signaling pathway (GO: 0038194), Negative regulation of meiotic cell cycle (GO: 0051447), and Growth plate cartilage chondrocyte proliferation (GO: 0003419) (S8 Table).

**Fig 2:**
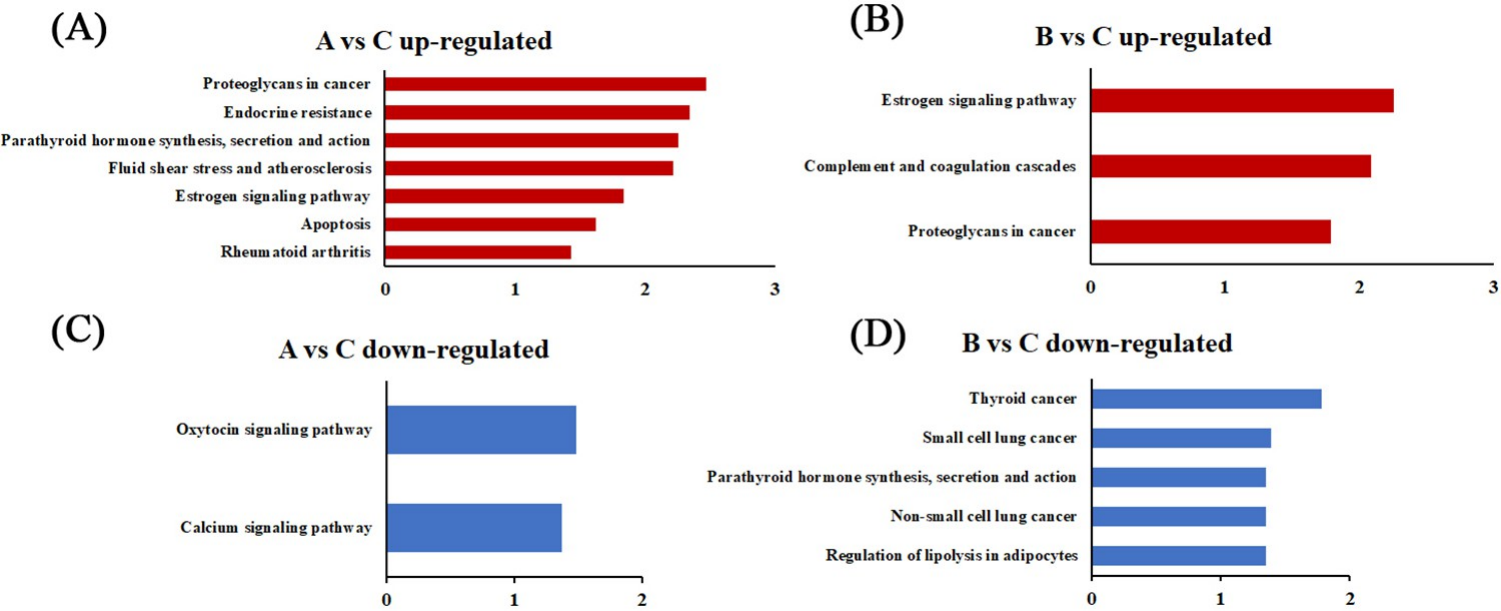
Pathway analysis of DEGs. A. KEGG enrichment analysis of upregulated genes for chronic low-temperature acclimation group (-log_10_ (p-value)>1.3). B. KEGG enrichment analysis of downregulated genes for chronic low-temperature acclimation group (-log_10_ (p-value)>1.3). C. KEGG enrichment analysis of upregulated genes for acute short cold stress group (-log_10_ (p-value)>1.3). D. KEGG enrichment analysis of downregulated genes for acute short cold stress group (-log_10_ (p-value)>1.3).

In KEGG pathway analysis, Proteoglycans in cancer and Estrogen signaling pathways were the common pathways among the significantly upregulated mRNAs for chronic low-temperature acclimation group and acute short cold stress group (Fig 2A and 2B). The pathways for downregulated mRNAs in chronic low-temperature acclimation group were Oxytocin signaling pathway and Calcium signaling pathway (Fig 2C), whereas Thyroid cancer, Small cell lung cancer, and Regulation of lipolysis in adipocytes were the significant pathways for downregulated mRNAs in acute short cold stress group (Fig 2D).

These results showed that under either chronic low-temperature acclimation or acute short cold stress, only a very few metabolic pathways were affected. Thus, the cold stress did not seriously affect skeletal muscle metabolism in Min pigs. Under acute short cold stress, only immune, endocrine, and cancer related pathways were affected. However, if the duration of exposure to low temperature was prolonged, apoptosis and calcium transduction were induced.

### LncRNAs are differentially expressed in the skeletal muscle of Min pigs in a cold environment

**Fig 3:**
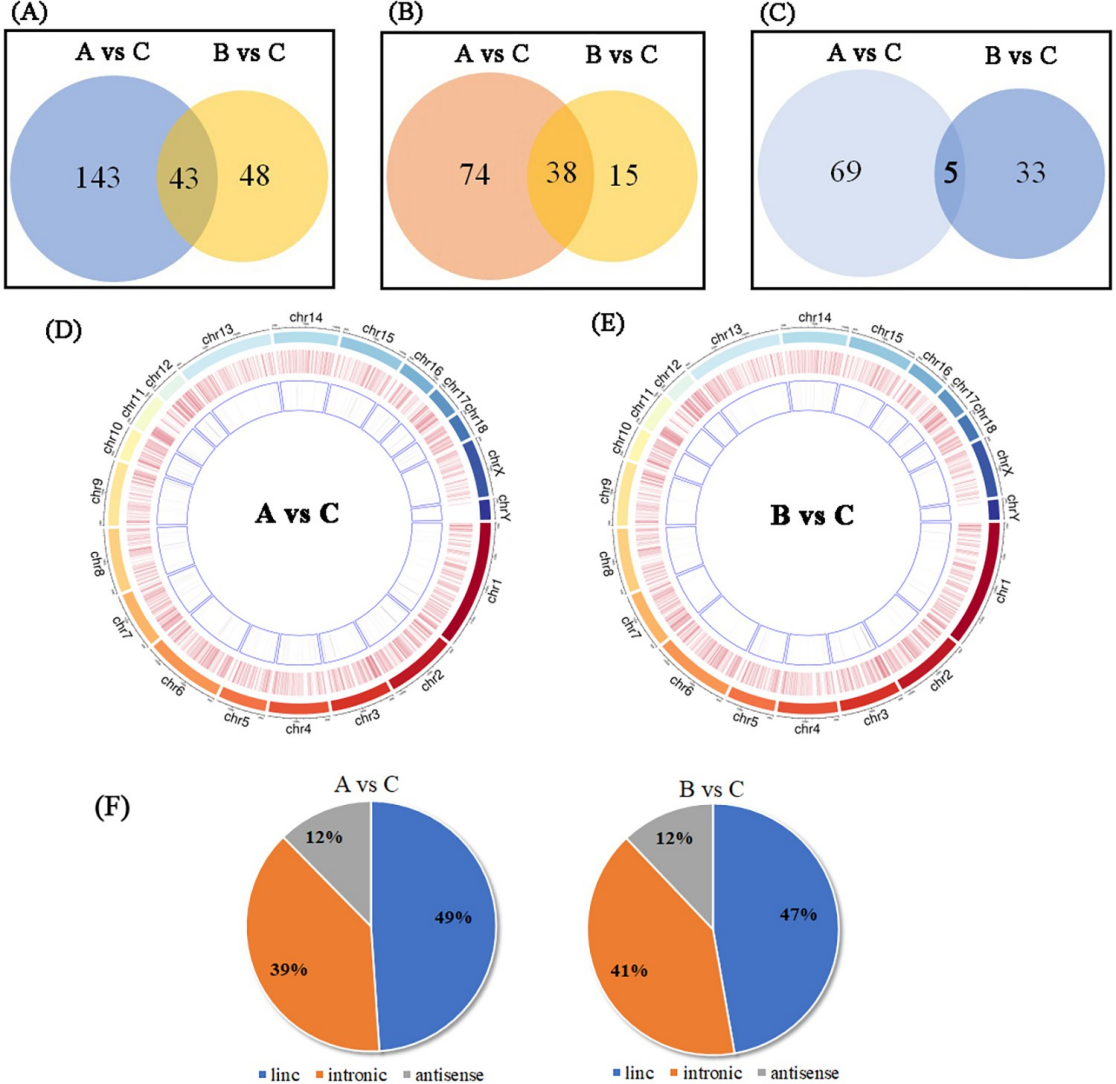
Differentially expressed lncRNAs in the skeletal muscle of the Min pig under cold stress. A. Total differentially expressed lncRNAs in the chronic low-temperature acclimation group and acute short cold stress group (log_2_FC >1 and q < 0.05). B. Upregulated differentially expressed lncRNAs in the chronic low-temperature acclimation group and acute short cold stress group. C. Downregulated differentially expressed lncRNAs in the chronic low-temperature acclimation group and acute short cold stress group. D. The distribution of lncRNAs on pig chromosomes for chronic low-temperature acclimation group. The maximum circle represents the chromosome length of the pig genome; the second circle represents all lncRNAs detected in this experiment, and the smallest circle represents the significantly differentially expressed lncRNAs (|log _2_FC|>1 and q<0.05). E. The distribution of lncRNAs on pig chromosomes for acute short cold stress group. The means of every circle is same to D. F. The proportions of three categories lncRNAs for chronic low-temperature acclimation group and acute short cold stress group.

LncRNAs usually play important roles at pre-transcriptional, transcriptional, and post-transcriptional levels. However, their roles in skeletal muscle physiology under cold conditions are unclear. Significantly differentially expressed lncRNAs in chronic low-temperature acclimation group and acute short cold stress group were identified by RNA-seq (p value < 0.05) (S9 and S10 Tables). The top 10 upregulated and the top 10 downregulated lncRNAs in the two groups are listed in S11 and S12 Tables. In particular, 43 lncRNAs were commonly differentially expressed in two groups, with 38 upregulated and 5 downregulated. There were 143 differentially expressed lncRNAs that were particular in chronic low-temperature acclimation group and 48 differentially expressed lncRNAs that were particular in acute short cold stress group (Fig 3A–3C). The general change trend of lncRNAs was roughly the same as with mRNA. With prolonged cold stress, more changes in lncRNAs occurred. These significantly differentially expressed lncRNAs were relatively uniform distributed on chromosomes (Fig 3D and 3E). Based on their position on the chromosome, the lncRNAs were classified into three categories: linc, intronic, and antisense. Proportion of each category is calculated and showed in Fig 3F. As shown, linc lncRNAs accounted for the highest proportion in both treatment group.

### The co‐expression network of lncRNA‐mRNA and differentially expressed lncRNAs functional prediction

**Fig 4:**
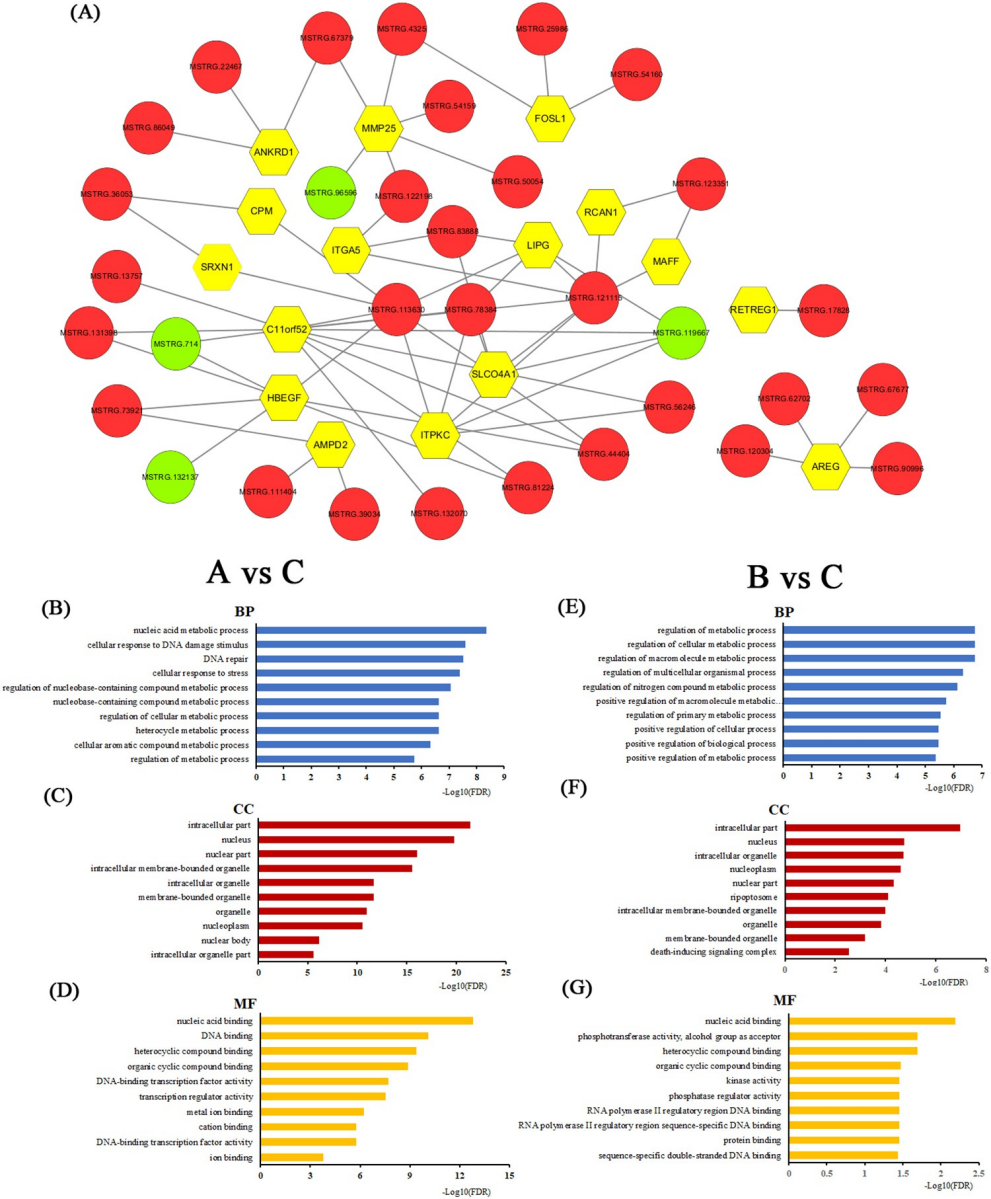
lncRNA-mRNA-network analysis. A. Blue nodes represent upregulated mRNAs; red nodes represent upregulated lncRNAs, and green nodes represent downregulated lncRNAs. B, C, and D illustrate GO analyses of target mRNAs of differentially expressed lncRNAs of the chronic low-temperature acclimation group. E, F and G illustrate GO analyses of target mRNAs of differentially expressed lncRNAs of the acute short cold stress group.

As shown in Fig 4A, the co-expression network profile was constructed, which consisted of 49 network nodes and 65 connections among 16 intersecting upregulated mRNAs and 33 differentially co-expressed lncRNAs. There were four negative and 61 positive interactions within the network. Moreover, our data showed that one mRNA may be linked with 1–11 lncRNAs, and that one lncRNA may be linked with 1–7 mRNAs.

To predict the functions of the lncRNAs, we first identified and then conducted a functional enrichment analysis of the mRNAs co-expressed with each of the differentially expressed lncRNAs. The enriched functional terms were used as the predicted functional terms for each given lncRNA. As shown in Fig 4B–4D, the GO analysis indicated that the most enriched GO terms targeted by the mRNAs co-expressed with lncRNAs for the A vs. C comparison were nucleic acid metabolic process (ontology: biological process, GO:0090304), intracellular part (ontology: cellular component, GO:0044424) and nucleic acid binding (ontology: molecular function, GO:0003676). The most enriched GO terms targeted by the mRNAs co-expressed with lncRNAs for the B vs. C comparison (Fig 4E–4G) were regulation of metabolic process (ontology: biological process, GO:0019222), intracellular part (ontology: cellular component, GO:0044424) and nucleic acid binding (ontology: molecular function, GO:0003676).

Furthermore, the KEGG pathway analysis indicated that the mRNAs co-expressed with lncRNAs of A vs. C groups were involved in the Herpes simplex virus 1 infection (q-value = 0.000026), MAPK signaling pathway (q-value = 0.0131) and Fanconi anemia pathway (q-value = 0.0466). At the same time, the KEGG pathways for the B vs. C groups were involved in the MAPK signaling pathway (q value = 0.0045) and the TNF signaling pathway (q value = 0.0073). From this it can be seen that MAPK signaling pathway was the primary response pathway.

### Validation of lncRNAs and mRNAs

**Fig 5:**
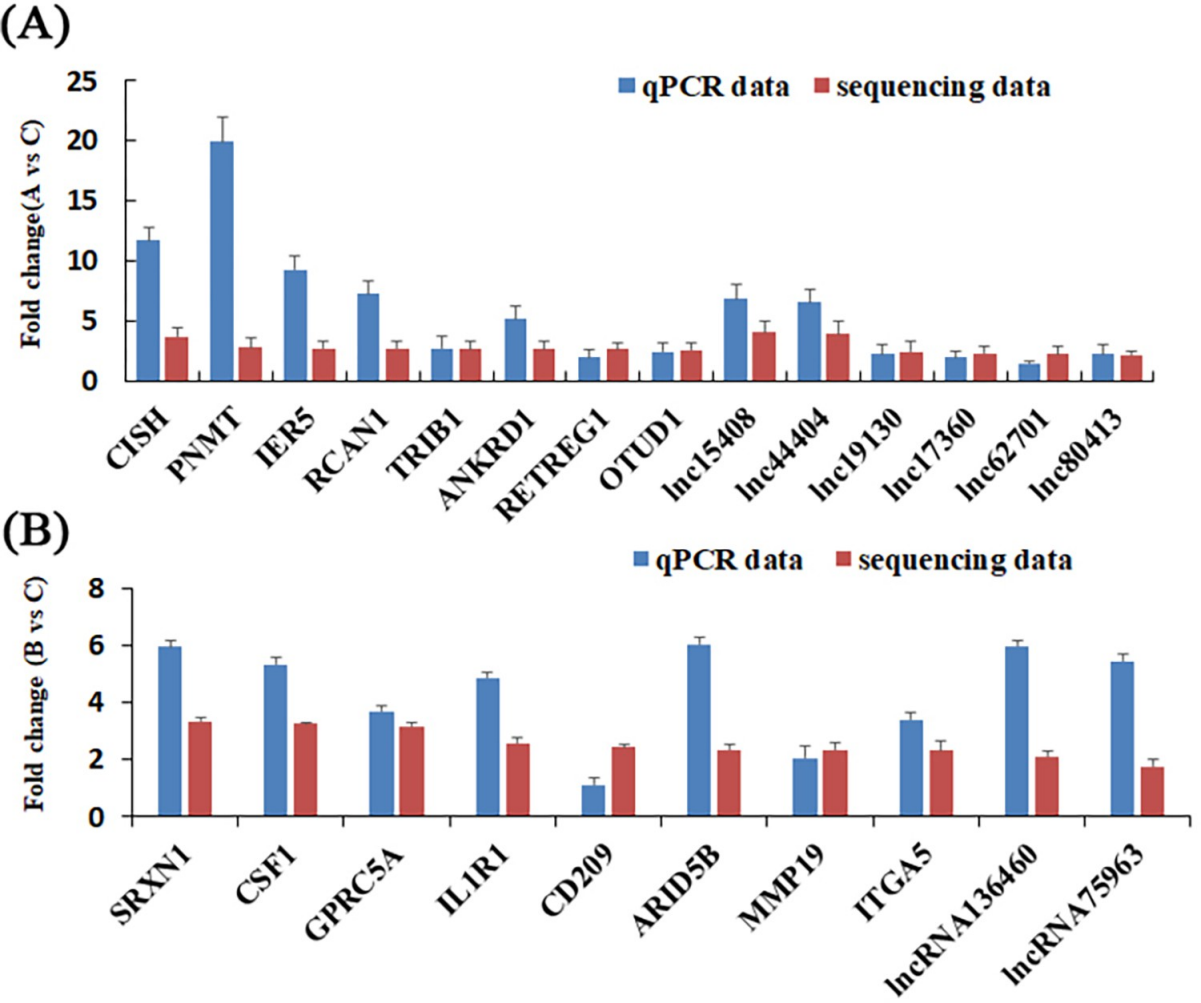
The differential expression of lncRNAs and mRNAs validated by quantitative real-time PCR (qRT-PCR). A. The qRT-PCR and RNA-seq results of chronic low-temperature acclimation group. B. The qRT-PCR and RNA-seq results of acute short cold stress group.

For verification of the RNA‐seq results, 10 mRNAs and six lncRNAs involved in the A vs. C group comparison and eight mRNAs and two lncRNAs involved in the B vs. C group comparison were selected and verified by qRT‐PCR in skeletal muscle from Min pigs. The results revealed that the variation tendency of mRNA and lncRNA from RNA-seq were coincident with the qRT-PCR results. This showed that the results of RNA-seq were accurate and reliable (Fig 5).

## Discussion

Studies in neonatal mammals and adult rodents have shown that brown adipose tissue (BAT) is an important site for non-shivering thermogenesis (NST) [15]. As a result, most of the research on non-shivering thermogenesis in the past two decades has focused on brown fat. Although BAT plays an important role in many mammals, it is usually limited to the neonatal stage and has a diminished role in adult large non-hibernating mammals, including humans. In addition, there are some warm-blooded animals, such as birds, marsupials, and pigs, that can maintain a constant Tc without BAT and that live in cold climates [16–18]. Therefore, it has been proposed that skeletal muscle is another important site of non-shivering thermogenesis [19].

A rigorous cryogenic experiment should be performed under temperature-controlled conditions, such as in previous studies of mice [20] and fish [21]. However, it is difficult to achieve accurate temperature control for large mammals such as pigs, cattle, or wild animals. Therefore, low-temperature treatment under natural conditions is generally used. For example, in beef cattle, through measuring the ambient temperature during the test period, three total cold load (TCL) classes were defined as less than −5°C (mild cold stress), −15°C (moderate cold stress), and −25°C (severe cold stress) [22]. Tibetan sheep are a species of antelope that live on the Qinghai–Tibet Plateau at an altitude of 3,300 meters. Samples collected in June were defined as the warm group, and samples collected in December were defined as the cold group [23]. The Min pig is a breed that is not very sensitive to temperature. With reference to the definition standard of beef cattle, and considering that pigs are smaller than cattle, we defined severe cold stress as a temperature below −20°C. The pig farm was situated at 125°42’ E longitude and 44°04’ N latitude. This area has a temperate continental monsoon climate. The maximum temperature in early November is 5°C, and by the end of December the maximum temperature will drop to −18°C. The minimum temperature will also drop from −3°C to −24°C. The chronic low-temperature experiment was carried out in November and December.

The emergence of next-generation sequencing technology allows researchers to perform quantitative and qualitative analyses at the whole transcriptome level, thereby improving the efficiency and accuracy of the analysis. In this study, the technique was used to analyze the skeletal muscle of Min pigs subjected to chronic low-temperature acclimation (the A group) and acute short cold exposure (the B group). Compared to the B group, greater transcriptional responses were observed in the A group. It is possible that A group individuals experienced greater physiological stress. More upregulated mRNAs were found in both groups compared to downregulated mRNAs. This finding agrees with those of a study in killifish, where cold acclimation at 5°C induced 5,460 upregulated genes compared with 1,746 downregulated genes in muscle transcriptomes [24]. Similar results were found in spotted seatrout [25].

Sixteen genes having been previously functionally annotated were upregulated in the two treatment groups (the A and B groups). They primarily focused on oxidative stress (*SRXN1*, *MAFF*) [26,27], immune and inflammatory responses (*ITPKC*, *AREG*, *MMP25*, *FOSL1*) [28–31], the nervous system (*RETREG1*, *GADD45A*, *RCAN1*) [32–34], lipid metabolism (*LRP11*, *LIPG*, *ITGA5*, *AMPD2*) [35–38], solute transport (*SLC19A2*, *SLC28A1*, *SLCO4A1*), fertility (*HBEGF*) [39] and skeletal muscle fiber type conversion (*ANKRD1*) [40]. Three genes were downregulated in both groups: *RGS14*, *MTX3*, and *TMEM139*. *RGS14* can regulate post synaptic plasticity in neurons [41] and *TMEM139* is involved in substance transport [42].

In addition to these common upregulated genes, there were some specific upregulated genes in the A and B groups. In the A group, four genes related to the nervous system were significantly upregulated by more than five times. For example, the *NTSR2* gene with the highest upregulated expression level can make mice more sensitive to heat and pain [43]. The second was the *ARC* gene, a neuronal gene that is essential for durable information storage in the mammalian brain and that has been implicated in various neurological diseases [44]. There were eight genes of the solute carrier superfamily (SLC): *SLC2A4*, *SLC2A5*, *SLC4A10*, *SLC19A2*, *SLC20A1*, *SLC28A1*, *SLCO4A1*, and *SLC38A2* upregulated in A group. This family mediates the transmembrane transport of various solutes between cells and the outside world or within cells, regulates the transmembrane transport of basic chemicals (including water, nutrients, hormones, and many drugs), maintains intracellular homeostasis, and maintains cell membrane integrity [45]. At the cell surface, SLC proteins are viewed as gatekeepers of the cellular milieu, dynamically responding to different metabolic states [46]. As a number of SLC genes were upregulated, this suggests that long-term low-temperature stress altered the transport of basic chemicals in the cells.

In group B, the genes related to energy metabolism and stress were upregulated. The *HGFAC* gene, which was the most upregulated gene, plays a role in the repair and regeneration of damaged tissues and organs. The HGFAC-HGF-PPARγ signaling pathway participates in the regulation of systemic carbohydrate, glucose, and lipid metabolism [47]. The *TREH* gene, which was upregulated 5.59 times, is an enzyme that catalyzes the hydrolysis of trehalose and that has been confirmed to be affected by low-temperature stress in insects and plants [48,49]. The enzyme plays an important role in growth and recovery from injury. The *TRIB3* gene belongs to the Tribbles signal regulatory protein family and is a key “stress regulation switch” involved in biological processes such as the endoplasmic reticulum stress response, cell growth, and cell differentiation [50]. Acute and short cold exposure triggers a stress response in the body.

In this study, *SERCA1a* (ATPase sarcoplasmic/endoplasmic reticulum Ca2+ transporter 1), which is generally considered to play an important role in non-shivering thermogenesis [19], was not changed significantly, but the expression level of its regulator *SLN* was reduced by half. This is consistent with the findings of a study in birds [51]. It has been speculated that SLN-mediated non-shivering thermogenesis (NST) may not be an important mechanism of thermoregulation in pigs. Other widely recognized genes such as *CIRBP* (cold inducible RNA binding protein) and *HSP70* (heat shock protein 70), which play important roles in muscle non-shivering thermogenesis [52,53] were also not found to have significant changes in this study. This result indicates that the mechanism of skeletal muscle thermogenesis is very complex. Moreover, gene expression has significant temporal and spatial specificity and is affected by multiple factors such as species, age, sex, tissue, and the environment.

There were two biological pathways with common activation in the A and B groups. One was the estrogen signaling pathway, and the other was proteoglycans in cancer. It is widely recognized that cold stress extends the estrous cycle, thereby causing reproductive hormone disorders [54]. Our results were consistent with this, as we found that the estrogen signaling pathway was enriched. The estrogen signaling pathway is a temperature-sensitive pathway. It can be induced by not only cold stress but also by heat stress [55,56]. Estrogens are not only crucial in sexual maturation and reproduction but are also highly involved in a wide range of brain functions, and they play crucial roles in skeletal muscle homeostasis and exercise capacity [57,58]. Proteoglycans are major macromolecules of the extracellular matrix. This pathway affects broader aspects of cancer initiation and the progression process, including regulation of cell metabolism and immune supervision [59]. Proteoglycans in the cancer pathway were continually upregulated under the cold treatments. Although there is no direct evidence for proteoglycans functioning in cold stress, according to their known functions we speculated that they were involved in changes in cell metabolism.

Low temperature can reduce blood flow in muscles [60]. In Min pigs after acute short cold stress, the complement and coagulation cascades pathways were active, indicating that cells were damaged and tended to clot [61]. Once a microthrombus is formed, it will cause a circulatory disturbance, resulting in tissue necrosis and frostbite. Under the same low-temperature environment, large white pigs developed clear signs of frostbite, while Min pigs did not [14]. In the chronic low-temperature acclimation group, this pathway was no longer enriched. We speculated that with the prolongation of cold treatment, Min pigs reregulated the genes of this pathway to normal levels through an unknown mechanism. Compared to acute short cold stress, there were additional enriched pathways such as rheumatoid arthritis, apoptosis, fluid shear stress and atherosclerosis, parathyroid hormone synthesis, secretion and action, and endocrine resistance pathways. This means that the chronic low temperature did more damage to the body.

As is well known, lncRNAs do not encode proteins but rather influence numerous cellular processes [62]. At present, the lncRNAs database for pigs is still incomplete, and thus it is necessary to predict the potential functions of lncRNAs through the analysis of their target genes. As shown by the results, the change trend of lncRNAs was consistent with that for mRNA. The target genes of A and B groups regulated by differentially expressed lncRNAs were all enriched in the MAPK signaling pathway. MAPK (mitogen-activated protein kinase) signaling pathways are generally considered to be survival pathways for enhancing cold resistance in animals and plants [63,64]. These pathways are involved in various biological events, including metabolic reprogramming and cell proliferation, survival, and differentiation. In this experiment, lncRNAs of MSTRG.116126, MSTRG.121115, MSTRG.72468, MSTRG.11761, MSTRG.132686, MSTRG.16211, MSTRG.116129, MSTRG.40092, MSTRG.119667, MSTRG.78384, MSTRG.122198, and MSTRG.83888 regulated the *FOS*, *EPHA2*, *PLA2G4B*, *RASGRP1*, *CACNG8*, *MAP4K4*, and *IL1R1* genes in the MAPK pathway.

In addition to the MAPK pathway, the TNF signaling pathway was enriched under acute short cold stress. LncRNA of MSTRG.75963, MSTRG.40092, MSTRG.116129, MSTRG.54160, 96596, MSTRG.28484, MSTRG.40092, MSTRG.73921, MSTRG.39843, MSTRG.121115, MSTRG.122198, MSTRG.83888, MSTRG.119667, andMSTRG.121115regulated the*RIPK1*, *NFKBIA*, *FOS*, *ICAM1*, *IRF1*, *CFLAR*, *TNFRSF1B*, *CASP10*, *SOCS3*, *CSF1*, *IL15* and *MAP3K8* genes in the TNF pathway. Tumor necrosis factor (TNF) is a major mediator of apoptosis as well as inflammation and immunity, and it has been implicated in the pathogenesis of a wide spectrum of human diseases [65]. Activation of this pathway suggests an inflammatory response in skeletal muscle cells under cold stress.

## Conclusions

Our RNA-seq analysis demonstrated that cold exposure significantly altered gene expression in the Min pig. Sixteen common DEGs were identified in chronic low-temperature acclimation and acute short cold stress groups. These genes may be candidate genes for cold exposure biomarkers. A large number of special DEGs were identified in pigs that were exposed to control, acute short, and chronic low temperatures. DEGs were enriched in the estrogen signaling pathway and the proteoglycans in cancer pathway. The change trend of the screened differentially expressed lncRNAs was consistent with that of mRNAs, and the upregulated transcripts numbered more than the downregulated transcripts after cold exposure. The target genes regulated by these DEGs were enriched in the MAPK pathway under different temperature conditions. By identifying genes, lncRNAs, and cellular pathways involved in cold response, we have provided important insights for future strategies to analyze low-temperature tolerance in large mammals.

## Supporting information

S1 Fig The highest and lowest temperature of every day during the experiment. (TIFF)

S2 Fig The heat map component analysis results of differential genes. A. The heat map component analysis results of differential genes of chronic low-temperature acclimation group. B. The heat map component analysis results of differential genes of acute short cold stress group.
(TIFF)

S1 Table Primer sequence for qRT-PCR. (XLSX)

S2 Table Data filtering statistical analysis table. (XLSX)

S3 Table The differentially expressed mRNA between A vs C group. (XLSX)

S4 Table The differentially expressed mRNA between B vs C group. (XLSX)

S5 Table Go analysis of differentially expressed up-regulated genes in A vs C. (XLSM)

S6 Table Go analysis of differentially expressed up-regulated genes in B vs C. (XLSX)

S7 Table Go analysis of differentially expressed down-regulated genes in A vs C. (XLS)

S8 Table Go analysis of differentially expressed down-regulated genes in B vs C. (XLSX)

S9 Table The differentially expressed lncRNA in A vs C group. (XLSX)

S10 Table The differentially expressed lncRNA in B vs C group. (XLSX)

S11 Table Top 10 upregulated and downregulated lncRNAs in chronic low-temperature acclimation group. (XLSX)

S12 Table Top 10 upregulated and downregulated lncRNAs in acute short cold stress group. (XLSX)
