# Supplementary figures and images for: Min pig skeletal muscle response to cold stress
Source: PLoS One. 2022 Sep 26;17(9):e0274184. doi: 10.1371/journal.pone.0274184 (PMC9512212; doi:10.1371/journal.pone.0274184)

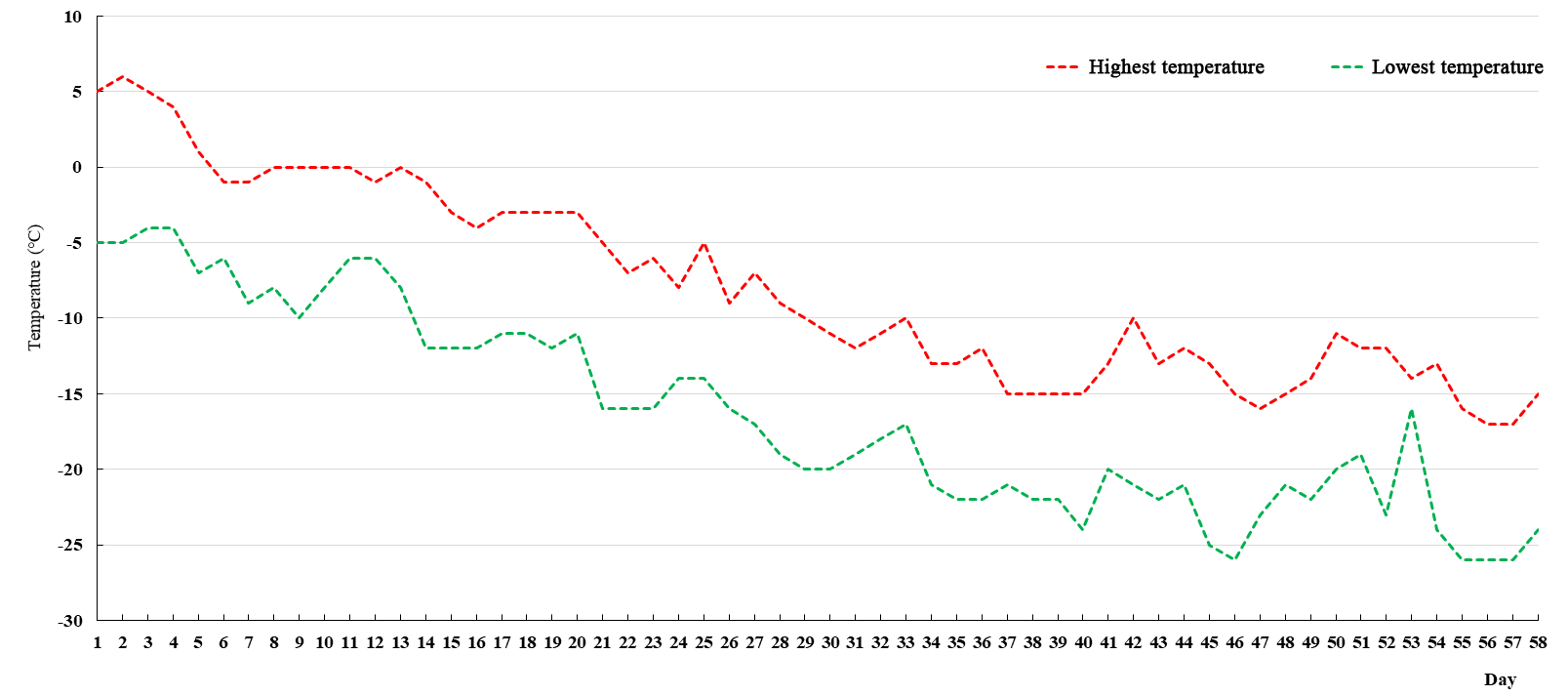

Supplement: S1 Fig — (TIFF) [file pone.0274184.s001.tiff]

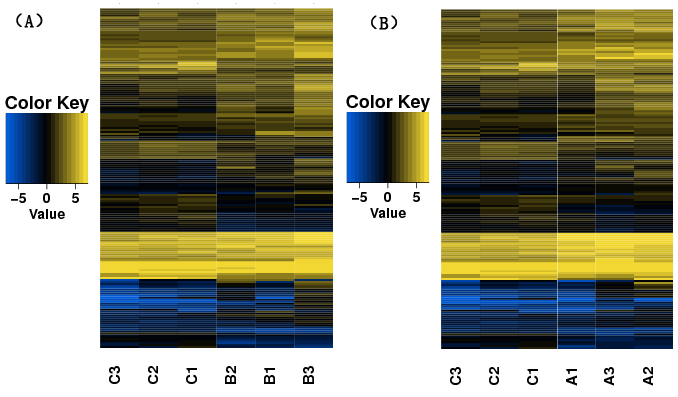

Supplement: S2 Fig — A. The heat map component analysis results of differential genes of chronic low-temperature acclimation group. B. The heat map component analysis results of differential genes of acute short cold stress group. (TIFF) [file pone.0274184.s002.tiff]
